# Nanogenerator-based self-powered sensors for data collection

**DOI:** 10.3762/bjnano.12.54

**Published:** 2021-07-08

**Authors:** Yicheng Shao, Maoliang Shen, Yuankai Zhou, Xin Cui, Lijie Li, Yan Zhang

**Affiliations:** 1 School of Physics, University of Electronic Science and Technology of China, Chengdu 610054, China; 2 College of Chemistry and Chemical Engineering, Center on Nanoenergy Research, School of Physical Science and Technology, Guangxi University, Nanning 530004, China; 3 Beijing Institute of Nanoenergy and Nanosystems, Chinese Academy of Sciences, Beijing 100083, China; 4 College of Nanoscience and Technology, University of Chinese Academy of Sciences, Beijing 100049, China; 5 Multidisciplinary Nanotechnology Centre, College of Engineering, Swansea University, Swansea, SA1 8EN, UK

**Keywords:** data collection, Internet of Things, nanogenerator, self-powered sensor, wearable device

## Abstract

Self-powered sensors can provide energy and environmental data for applications regarding the Internet of Things, big data, and artificial intelligence. Nanogenerators provide excellent material compatibility, which also leads to a rich variety of nanogenerator-based self-powered sensors. This article reviews the development of nanogenerator-based self-powered sensors for the collection of human physiological data and external environmental data. Nanogenerator-based self-powered sensors can be designed to detect physiological data as wearable and implantable devices. Nanogenerator-based self-powered sensors are a solution for collecting data and expanding data dimensions in a future intelligent society. The future key challenges and potential solutions regarding nanogenerator-based self-powered sensors are discussed.

## Introduction

Self-powered sensor systems can harvest and convert environmental energy to electricity, which enables sensor operation without external power source [1–2]. Nanogenerators (NGs) can effectively harvest energy various low-frequency mechanical motions from the environment. NG-based self-powered sensors act as data collection units for traffic [3–11], meteorological environment [12–21], human movement [22–27], viscera [28–30], body fluid composition [31–39], biological nerve impulses [40], and gas sensors [18–20]. Self-powered sensors based on NGs can analyze objects from a new perspective. The materials of NG come from a wide range of sources, such as wood [41–42], paper [43–46], waste milk carton [15], and skin [47–49]. Thus, low-cost self-powered sensors can be deployed on a large scale and are a good candidate for data sources for the Internet of things (IoT), big data, and artificial intelligence (AI).

NGs can be used as both pressure sensors and as energy supplies. Triboelectric nanogenerators (TENGs) were used as electronic skin for pressure detection and material identification [50–51]. Pressure sensors based on piezoelectric nanogenerators (PENGs) were used to detect tiny pressure deviations from water droplets [52–53], wind flow [53–56], or even human pulse waveforms [57–58]. NG-based self-powered sensors can be applied in traffic monitoring [3,59–61], and road and bridge monitoring [4,59]. Data regarding the driving status of vehicles, the operating status of vehicle components, and the driver usage habits are related to the safety of vehicle driving and the experience of the driver [5–11].

The principle of operation of TENGs is the triboelectrification/contact electrification (CE) process [62–64]. TENGs have four working modes: the common vertical contact-separation mode, the single-electrode mode, the contact-sliding mode, and the freestanding-triboelectric-layer mode [2,65]. TENGs can be made of many different materials with low manufacturing cost, environmental friendliness, and low maintenance cost. TENG-based sensors can collect multidimensional and large-scale data, which are a novel data source for big data and AI. Especially, TENGs are good candidates for designing AI sensors [66]. TENGs can be used as an energy source for traditional sensors to collect tiny amounts of energy from the environment, such as from liquid droplets [67]. The performance of TENGs can be improved through material optimization and charge-accumulation strategies [4,62,64,68–69]. In addition, TENGs can be directly used as sensors. For example, TENGs can collect irregular and low-frequency wave energy to generate electricity from blue energy [70]. Self-powered sensors based on TENGs can collect hydrological data such as wave information [12–13], water quality [14–15], and ion concentration [16–17,71], which can be used for weather forecasting, disaster warning, and water quality protection. In addition, long-term monitoring and collection of hydrological data can also provide a certain reference for the design of sterilization and algae removal [72], wastewater treatment [73–74], and electrochemical corrosion protection of metal surfaces and battery cathodes [56,75–76]. TENG-based special flexible pressure sensors can be placed on the surface of human skin to monitor the physiological activities of the human body, such as joint bending, extension, and body rotation [22–26,77].

**Figure 1:**
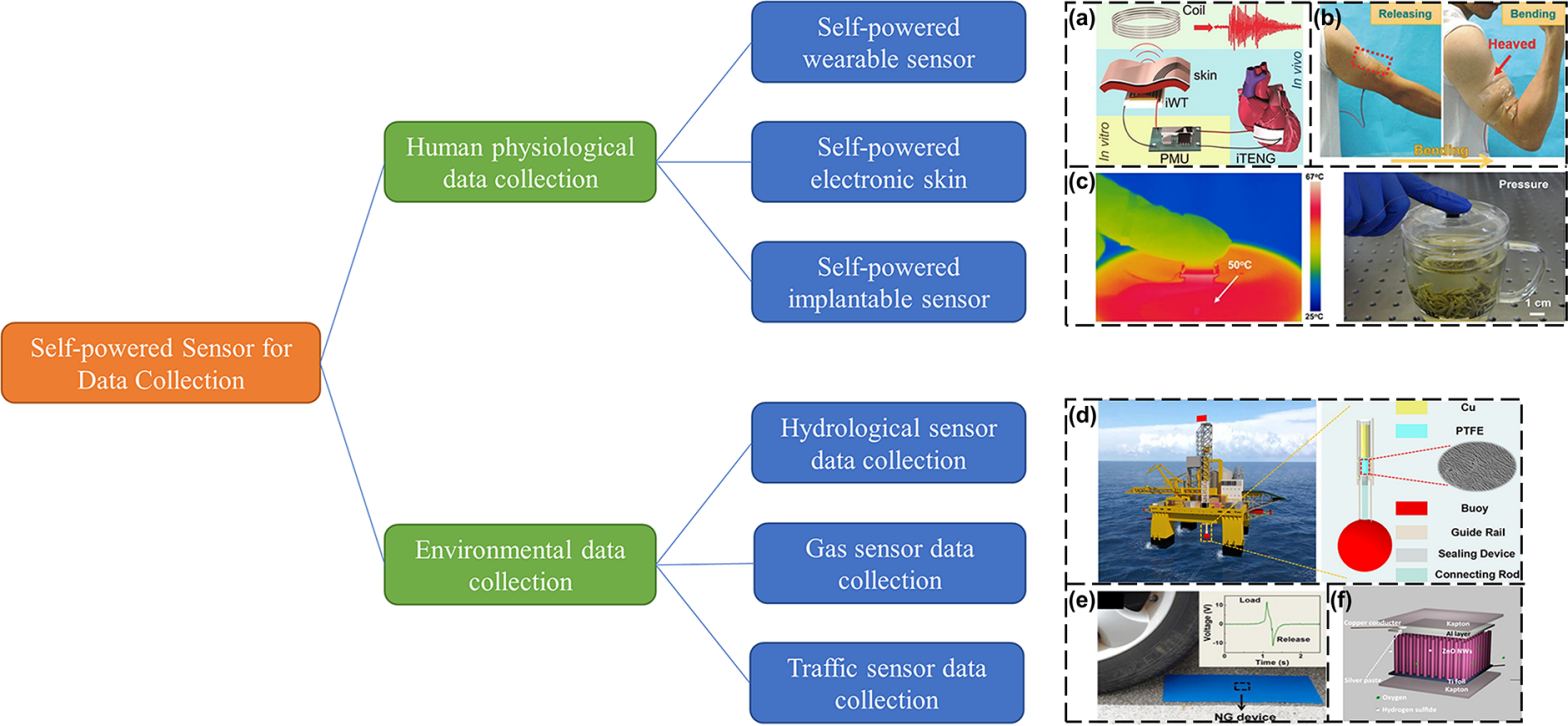
The development of nanogenerator-based self-powered sensors in the collection of human physiological data and of environmental data. Figure 1a was reproduced with permission from [28], Copyright 2016 American Chemical Society. Figure 1b is adapted from [22]. Copyright © 2018 WILEY-VCH Verlag GmbH & Co. KGaA, Weinheim. Used with permission from Zhen Wen et al., “TA Wrinkled PEDOT:PSS Film Based Stretchable and Transparent Triboelectric Nanogenerator for Wearable Energy Harvesters and Active Motion Sensors”, Advanced Functional Materials, John Wiley and Sons. Figure 1c is reproduced from [51] (© 2020 Yang Wang et al., some rights reserved; exclusive licensee American Association for the Advancement of Science. No claim to original U.S. Government Works. Distributed under a Creative Commons Attribution License 4.0 (CC BY) https://creativecommons.org/licenses/by/4.0/). Figure 1d reproduced with permission from [12], Copyright 2016 American Chemical Society. Figure 1e was reproduced from [3], Nano Energy, vol. 2, by L. Lin, Y. Hu, C. Xu, Y. Zhang, R. Zhang, X. Wen, Z. L. Wang, “Transparent flexible nanogenerator as self-powered sensor for transportation monitoring“, pages no. 75–81, Copyright (2012), with permission from Elsevier. Figure 1f was republished from [18] (X. Xue et al., “Surface free-carrier screening effect on the output of a ZnO nanowire nanogenerator and its potential as a self-powered active gas sensor”, Nanotechnology, vol. 24, no. 22, 225501, published on 30 April 2013, https://doi.org/10.1088/0957-4484/24/22/225501 © 2013 IOP Publishing Ltd. Publishing. Reproduced with permission via Copyright Clearance Center. All rights reserved.

Figure 1 shows the application NG-based self-powered sensors in the collection of human physiological and of environmental data. On this basis, developments and challenges of future NG-based self-powered sensors in data-driven intelligent systems are proposed.

## Review

### Human physiological data collection based on self-powered sensors

#### Self-powered wearable sensors and electronic skin

**Figure 2:**
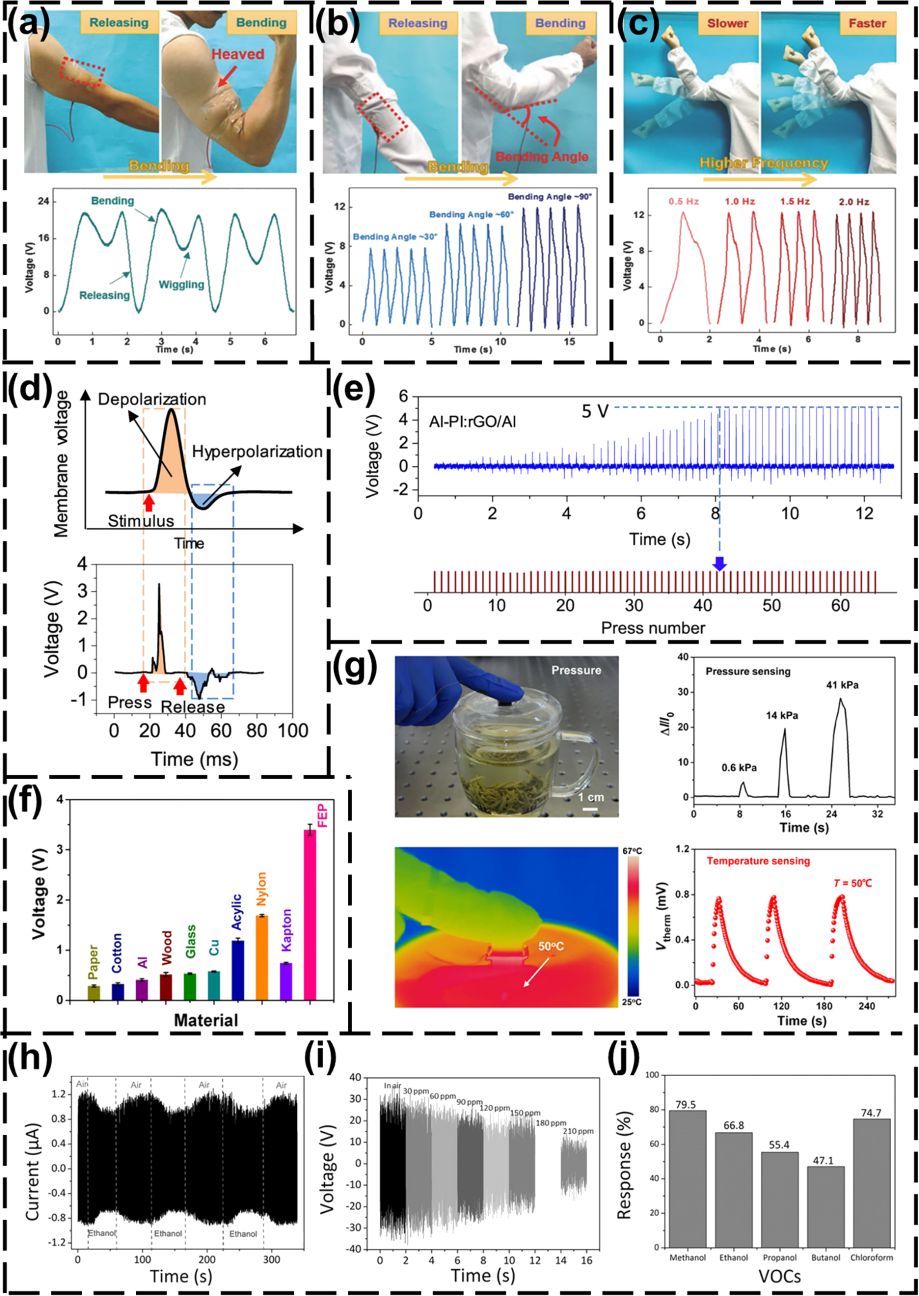
NG-based self-powered wearable sensor and electronic skin for data collection. (a) A schematic diagram of a WP-TENG-based motion sensor installed on the arm to detect the motion state, and the voltage signal generated by sensor when the arm is bent and released [22]. Figure 2a–c was reproduced from [22] (for permission, please see the caption of Figure 1b). (b) The sensor is installed on the elbow to detect different bending angles of the elbow. Voltage signals of different magnitudes are generated when the elbow is bent at different angles [22]. (c) The sensor is installed on the elbow. The elbow is bent and released at different frequencies and the voltage signals are generated at different frequencies [22]. (d) The biologic potential generated by external pressure and an intelligent neuromorphic sensor [90]. Figure 2d and Figure 2e were reprinted with permission from [90], Copyright 2020 American Chemical Society. (e) The output of the sensor changing with the increase of pressure times [90]. (f) The output voltage generated when in contact with different materials [51]. Figure 2f and Figure 2g were reproduced from [51] (for permission, please see the caption of Figure 1c) (g) A multi-functional self-powered sensor detects pressure and temperature and generates output signals [51]. (h) The output current of smelling electronic skin exposed to air and ethanol at a concentration of 60 ppm [91]. Figure 2h–j was reproduced from [91]. Copyright © 2016 WILEY‐VCH Verlag GmbH & Co. KGaA, Weinheim. Used with permission from Xinyu Xue et al., “Outputting Olfactory Bionic Electric Impulse by PANI/PTFE/PANI Sandwich Nanostructures and their Application as Flexible, Smelling Electronic Skin”, Advanced Functional Materials, John Wiley and Sons. (i) The output voltage of smelling electronic skin as the ethanol concentration increases [91]. (j) The response of smelling electronic skin to several volatile organic compounds [91].

Self-powered wearable sensors to collect human motion data can provide a data set for medical diagnosis and rehabilitation, sports training, human motion recognition, respiratory monitoring, and human 3D motion modeling [78–80]. These data can be used for real-time detection of human health or human–computer interaction [81–82]. Wen et al. [22] manufactured a transparent and stretchable wrinkled (maximum strain approximately 100%) TENG (WP-TENG) based on a poly(3,4-ethylenedioxythiophene):poly(4-styrenesulfonate) (PEDOT:PSS) electrode and installed the WP-TENG-based self-powered motion sensor at different positions of a human arm. The WP-TENG was placed on the skin above the muscles of the arm, as shown in Figure 2a. When the arm is bent, the muscles stretch the sensor to a larger contact area, and a voltage variation is generated by the sensor. An output voltage of about 23 V is generated. When the arm is released, the voltage returns to zero. The peak voltage varies with the bending angle of the elbow, as shown in Figure 2b, and the frequency of joint motion, as shown in Figure 2c. The self-powered motion sensor can obtain the bending angle of the elbow joint through the peak voltage output, and monitor the motion frequency in real time by counting the peaks. Furthermore, self-powered motion sensors can be used for gesture recognition [78,83–84]. The combination of a self-powered motion sensor and a back-end data processing system based on machine learning (ML) can realize sign language recognition for people with language impairment. Zhou et al. [84] fabricated a stretchable sensor for sign language recognition. The stretchable sensor is installed on a glove by using a flexible material. When the fingers move, each finger generates an electrical signal. These signals are classified by using ML algorithms to obtain the text, and finally the text is converted into speech output. The method of ML processing uses a principal component analysis (PCA) algorithm for feature extraction and a support vector machine (SVM) algorithm for gesture recognition, with a recognition accuracy of 98.63% and recognition time of less than 1s. The front-end sensor could be replaced by a more advanced self-powered pressure/touch sensor based on PENGs/TENGs, which combined with back-end ML technology, can help disabled people to live and communicate normally. Self-powered motion sensors can also collect the weak mechanical energy generated in other physiological activities of the human body, such as heartbeat, breathing, and vocal cords [85–86]. Without external power supply, the back-end data processing technology can realize real-time detection and early warning of human health [87]. For example, voice can be recognized by vocal cord vibration, which can be recorded with a biosensor attached to the skin of the throat. Voice print recognition and speech recognition can be realized by employing back-end data processing technology [88].

PENG/TENG-based self-powered sensors are flexible and highly sensitive, which can reduce inconveniences of a sensor system during physical training [41]. Back-end intelligent analysis technology can be used to quickly detect the large amount of data collected to reconstruct muscle movement and accurately obtain exercise habits. For example, the movements of athletes can be detected and identified in real time using AI classification techniques [89]. Big data analysis technology can monitor the training status of athletes in real time and provide training suggestions [83].

TENGs have high sensitivity, and a slight strain can cause the output signal of TENGs to change. Self-powered sensors based on TENGs are feasible signal monitoring sensors for facial activity, breathing, vocal cord vibration, heartbeat, and other small physiological activities. Small physiological signals, such as facial activity data, can be used for monitoring the driver status to prevent non-hazardous driving and improve driving safety. In 2018, Meng et al. [10] proposed a TENG-based self-powered pressure sensor that can detect situations such as the driver stepping on the accelerator or blinking. In 2020, Lu et al. [11] further proposed a transparent stretchable self-powered sensor based on a polyacrylamide TENG (PL-TENG), which is used to detect driver fatigue and distraction while driving and then to alert the driver. Different actions of the driver, such as winking, opening mouth, nodding, and turning neck will cause the PL-TENG to output electrical signals with corresponding features. The output voltage can be processed by a recurrent neural network (RNN) and judged by multiple sensors to improve the driving safety. The TENG-based self-powered pressure sensor is more sensitive, more stable, and less costly than the near-infrared illuminator, with far-reaching implications for traffic safety.

TENG-based electronic skin has more functions. Inspired by the plasticity of human skin nerve signals, the researchers proposed a single-electrode TENG (SE-TENG) as an intelligent neuromorphic sensor using reduced graphene oxide [90]. Reduced graphene oxide can act as electronic trap, and the output information of the sensor contains real-time stimulation information and information about previous stimulations. The sensor can generate voltage pulses similar to biological mechanoreceptors under external pressure, as shown in Figure 2d. The upper panel of Figure 2d is the biological action potential, and the lower panel of Figure 2d is the voltage pulse generated by the sensor. The output monotonously increases with pressure. The output voltage reaches a saturation value after a certain number of sensor actuations, as shown in Figure 2e. This intelligent neuromorphic sensor that mimics synaptic enhancement and memory can be used as a human skin tactile sensing solution, providing rich data for artificial intelligence. Wang et al. proposed a hydrophobic polytetrafluoroethylene film and sponge-like graphene/polydimethylsiloxane composite material to prepare a multifunctional self-powered sensor [51]. The sensor can infer the performance of the material through the difference in the output of the electrical signal generated by contact with different materials, as shown in Figure 2f. The sensor can also measure pressure and temperature as shown in Figure 2g.

Electronic skin can also collect composition information of substances in the air. Xue et al. proposed a TENG-based smelling electronic skin that generates electrical impulses when exposed to gas flow or pressure [91]. The material of the electronic skin can react with volatile organic compounds (VOCs) in the air, such as ethanol, as shown in Figure 2h. In addition to detecting whether the air contains VOCs, the output open-circuit voltage of the smelling electronic skin is also negatively correlated with the concentration of the VOCs, as shown in Figure 2i. The smelling electronic skin responds to a variety of VOCs, such as methanol, propanol, butanol, and chloroform, as shown in Figure 2j.

#### Self-powered implantable sensors

**Figure 3:**
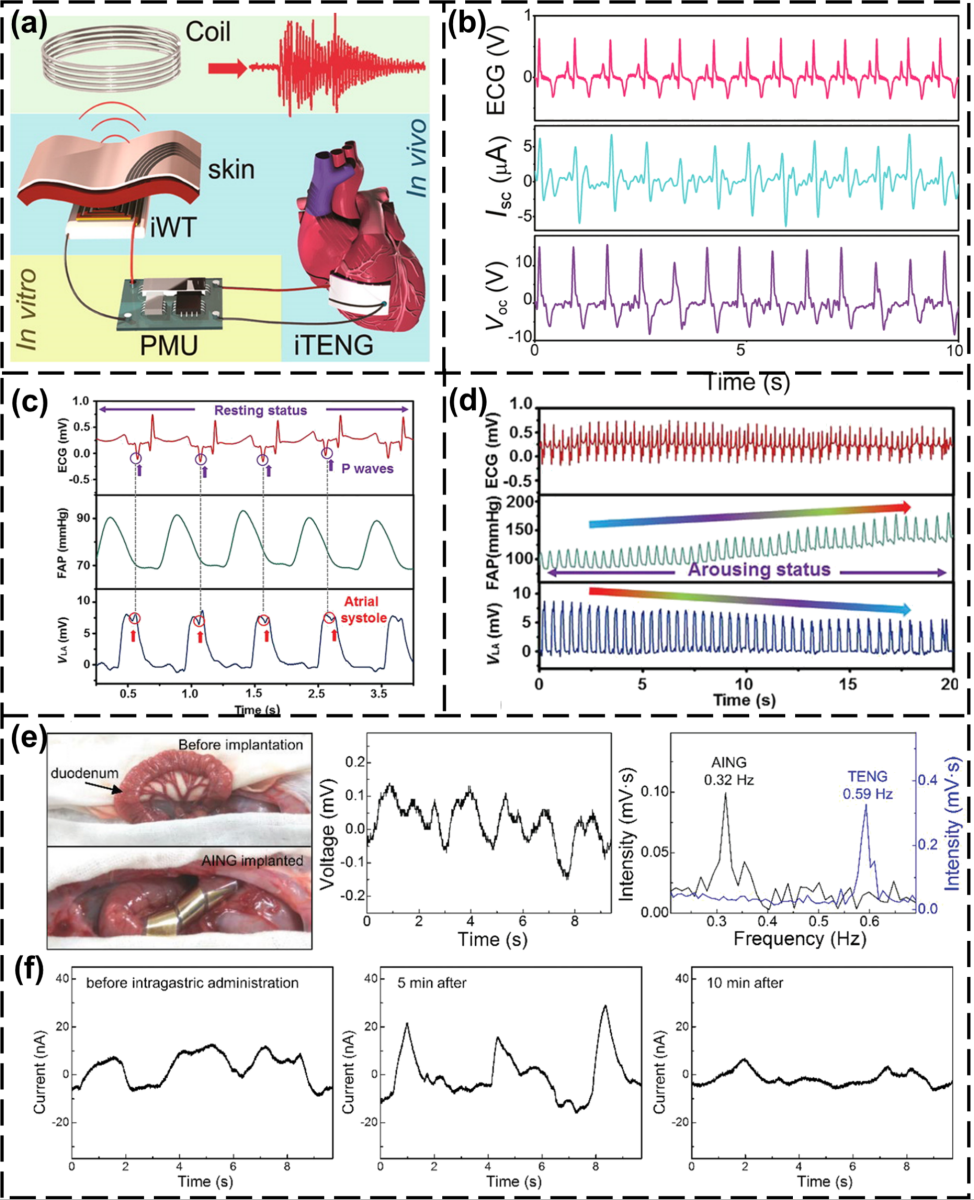
Self-powered implantable sensors in vivo. (a) Structure of heart data sensor based on an iTENG, implantable wireless transmitter (IWT) and power management unit (PMU) [28]. (b) Comparison of the output of the iTENG and ECG signal [28]. Figure 3a,b was reproduced with permission from [28] (for permission, please see the caption of Figure 1a). (c) Correspondence between ECG, FAP, and SEPS output signals [29]. Figure 3c,d was reproduced from [29]. Copyright © 2018 WILEY‐VCH Verlag GmbH & Co. KGaA, Weinheim. Used with permission from Zhou Li et al., “Transcatheter Self‐Powered Ultrasensitive Endocardial Pressure Sensor”, Advanced Functional Materials, John Wiley and Sons. (d) The change of ECG, FAP, and SEPS output voltage after epinephrine injection [29]. (e) When the ATNG is implanted in rabbits, the activity of the rabbit intestines causes changes in output signal of the ATNG [30]. Figure 3e,f were reproduced from [30]. Copyright © 2020 WILEY-VCH Verlag GmbH & Co. KGaA, Weinheim. Used with permission from Zhong Lin Wang et al., “Mechanically Asymmetrical Triboelectric Nanogenerator for Self ‐Powered Monitoring of In Vivo Microscale Weak Movement”, Advanced Functional Materials, John Wiley and Sons. (f) The electrical signal generated by the activity of the intestine after glucose injection under anesthesia [30].

Physiological data can be used for health monitoring. When analyzing the components in body fluids, such as glucose, external sensors will lose accuracy due to interference from other components in body fluids [92]. An internal blood glucose sensor would be more reliable. Given the many possible TENG materials, materials with good biocompatibility can used for implantable self-powered physiological sensors. The sensors can be powered by the mechanical energy from the biological activity of the organism, and provide physiological data. An implantable heart monitoring sensor based on a TENG can work stably for a long time, providing a solution for long-term heart monitoring. Zheng et al. proposed an implantable TENG (iTENG) to realize wireless heart monitoring in vivo [28]. The iTENG is not cytotoxic. The packaging material of the iTENG showed good biocompatibility in mouse fibroblasts. The iTENG was implanted between heart and pericardium of a Yorkshire pig to monitor the heartbeat signal. The iTENG can be driven by heartbeat movement, collect the heartbeat signal and send it wirelessly to the data-receiving end outside the body, as shown in Figure 3a. The output voltage is synchronous with the electrocardiogram (ECG) signal, as shown in Figure 3b. The iTENG provided a stable electrical signal output for more than 72 h in this case.

The changes of endocardial pressure (EP) have important clinical significance for patients with impaired heart function. The self-powered endocardial pressure sensor (SEPS) converts the energy of blood flow into electrical energy in the heart, which is used to detect changes in EP in real time [29]. The SEPS was implanted in the left atrium of a Yorkshire pig. Figure 3c shows the output voltages during cardiac contraction and relaxation. The changes in the output voltage signal are completely synchronous with the changes in the femoral arterial pressure (FAP) and ECG signals. After epinephrine is injected, the FAP rises, while the peak value of the SEPS output voltage slowly decreases, as shown in Figure 3d. The opposite trends of the two are synchronous, so the output voltage of SEPS can be used as an indicator of epinephrine efficiency. The SEPS can accurately monitor the EP of the ventricle in real time. Through sudden changes in the EP, life-threatening arrhythmias can be detected in a timely manner.

Compared with the heart, the physiological activity of the gastrointestinal tract is weaker. Traditional TENGs are affected by the heart and respiration, and it is difficult to obtain accurate information on the physiological activity of the gastrointestinal tract. Cheng et al. proposed an asymmetrical and ultrasensitive TENG (ATNG), which can monitor tiny gastrointestinal movements and collect gastrointestinal data [30]. The ATNG was implanted into the abdominal cavity of a rabbit, and the duodenal peristalsis signal of the rabbit was monitored, as shown in Figure 3e. Comparison with the frequency-domain transform of the traditional TENG output signal implanted in the same position shows that the 0.58 Hz signal captured by the traditional TENG is a breathing signal. The ATNG can capture the gastrointestinal movement signal with the small 0.32 Hz intestinal movement signal and eliminate the interference of breathing. Under anesthesia, glucose solution was injected into the stomach of the rabbit. After 5 min, the gastrointestinal peristalsis becomes intense, and after 10 min, the peristaltic recovery becomes smooth (Figure 3f). It shows that the ATNG can monitor the weak activity of the gastrointestinal tract of a rabbit and can provide data on weak physiological activities.

**Figure 4:**
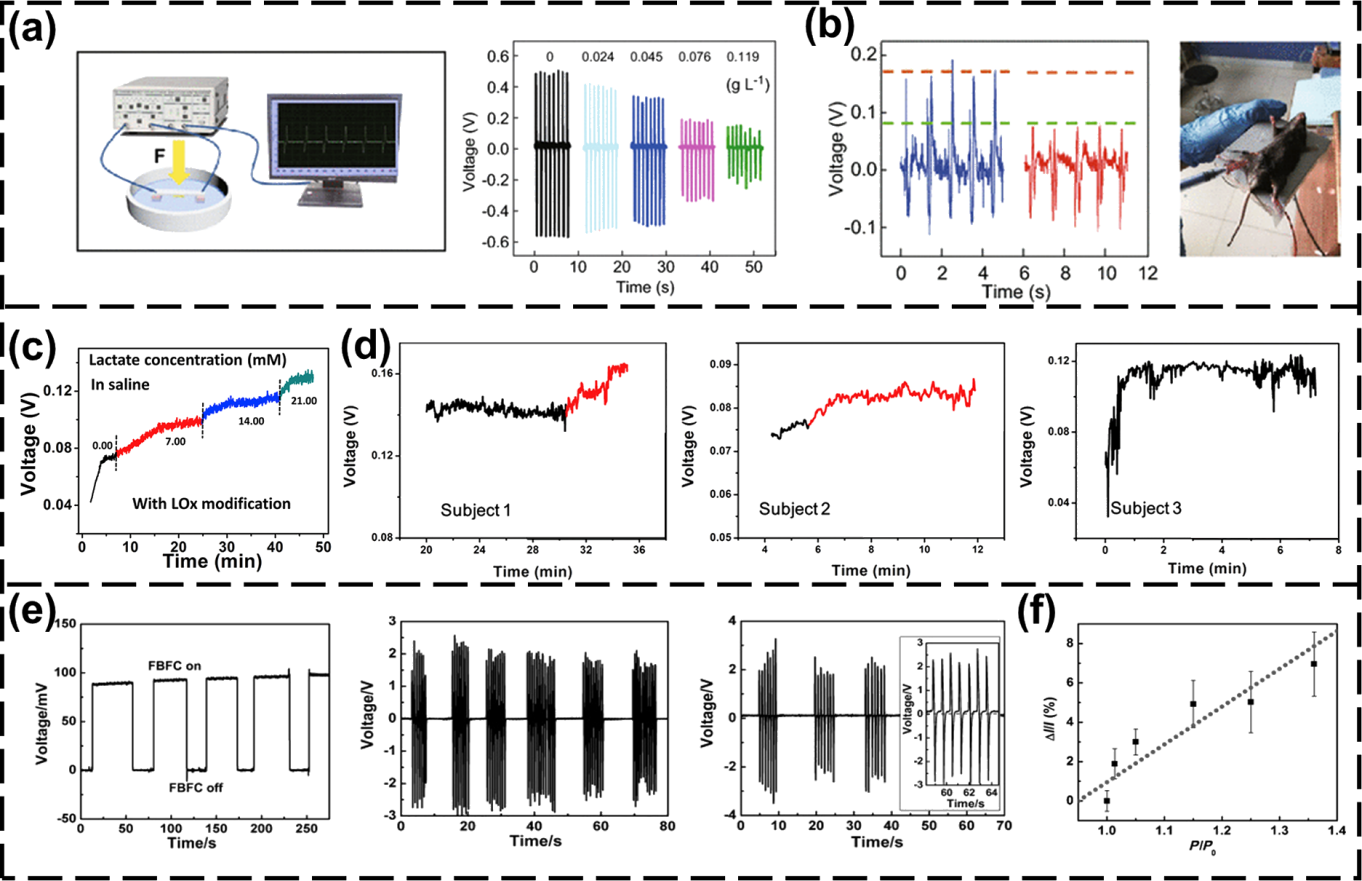
Structure and performance of self-powered body fluid sensors. (a) The system composition of the implantable skin-like glucometer and the voltage output in different concentrations of glucose [31]. Figure 4a,b was reproduced from [31] (© 2018 W. Zhang et al., published by Springer Nature, distributed under the terms of the Creative Commons Attribution 4.0 International License, https://creativecommons.org/licenses/by/4.0). (b) Implantable skin-like glucometer implanted in a mouse and the voltage output of the device under different blood glucose concentrations [31]. (c) The output voltage performance of sweat-evaporation-biosensing sensors in different concentrations of lactic acid [37]. Figure 4c,d was adapted from [37], Nano Energy, vol. 59, by H. Guan, T. Zhong, H. He, T. Zhao, L. Xing, Y. Zhang, X. Xue, “A self-powered wearable sweat-evaporation-biosensing analyzer for building sports big data”, pages no. 754–761, Copyright (2019), with permission from Elsevier. (d) Real-time voltage output generated by the sweat-evaporation-biosensing sensor when three people are cycling [37]. (e) The FBFC converts glucose to produce DC output, the FNG produces AC output from the same periodic pressure, and the hybrid system output of FBFC and FNG in series [35]. Figure 4e,f was reproduced from [35]. Copyright © 2011 WILEY‐VCH Verlag GmbH & Co. KGaA, Weinheim. Used with permission from Caofeng Pan et al., “Fiber‐Based Hybrid Nanogenerators for/as Self‐Powered Systems in Biological Liquid”, Angewandte Chemie International Edition, John Wiley and Sons. (f) Output of the hybrid system under different pressures [35].

An important indicator of human health is the composition of body fluids. The large amount of mechanical energy generated by the human body can also be used to drive a self-powered body fluid sensor to collect body fluid data [31–38]. In addition to the real-time detection of body fluids by self-powered technology, the self-powered technology can be used to deliver drugs to the patient according to changes in the chemical composition of the body fluids [93–94]. This self-powered experiential health management and treatment system is a development direction of future medicine. Zhang et al. [31] developed a self-powered implantable blood glucose meter based on the piezo-enzymatic-reaction coupling effect of GOx@ZnO (GOx: glucose oxidase) nanowires. By collecting the mechanical energy generated by human movement, the output voltage provided information about blood glucose concentration. As shown in Figure 4a, the output voltage of the self-powered blood glucose meter is inversely proportional to the glucose concentration. After implanting the device in a mouse, the output of the device changes with the blood glucose concentration, as shown in Figure 4b, which proves the feasibility of the device to collect blood glucose data.

Sweat data analysis is one of the directions to analyze the physical state of athletes. For example, lactic acid levels in the sweat of athletes are analyzed to determine whether they are tired and engaged in aerobic or anaerobic exercise. Guan et al. [37] proposed a self-powered analyzer that can detect changes in lactic acid in sweat. As shown in Figure 4c, the output voltage of the analyzer is proportional to the lactic acid concentration. The analyzer attached to the surface of human body uses the evaporation of sweat during human exercise to generate electricity, and its output performance is affected by the concentration of lactic acid in the sweat, as shown in Figure 4d. According to the change of output voltage data, big data analysis can be performed on the exercise status of the athlete. Training status and exercise intensity information can be obtained in real time. This is a new solution for big data sensing in sports. The mechanical energy generated by blood flow or body motion also can drive sensors for monitoring various indicators of body fluids. Pan et al. [35] proposed a self-powered blood pressure sensor that uses mechanical energy and biochemical energy. The fiber nanogenerator (FNG) and the fiber biofuel cell (FBFC) are fully integrated on a single carbon fiber. The FNG converts the periodically applied pressure in the liquid into an alternating current (AC) output, and the FBFC converts glucose in the blood into electrical energy to generate a direct current (DC) output. Figure 4e shows the output performance of FBFC and FNG, and the output performance of two devices in series. The output of FNG as a pressure sensor with pressure changes is shown in Figure 4f. The output is proportional to the pressure. By collecting mechanical energy and biochemical energy in the environment of the human body, this sensor can stably and continuously collect pressure data from human blood vessels and other body fluid environments. The NGs can be used for human health monitoring and blood pressure data analysis.

### Environmental data collection based on self-powered sensors

Environmental sensors can be used for collecting and processing data of electricity and gas maintenance, vehicle safety, and weather forecasting. The collection of environmental data requires real-time, long-term monitoring. Compared with traditional sensors, self-powered sensors based on PENGs/TENGs can convert mechanical energy into electricity. At the same time, they can also obtain environmental information. PENG/TENG-based self-powered sensors can be placed on roads and bridges, which can be powered by mechanical energy from traffic flow and bridge vibration. Self-powered sensors can be used to collect real-time data of vehicle speed, acceleration and tire status. Self-powered sensors can collect hydrological and meteorological data, providing powerful data tools for ambient intelligence in the future, such as improving driving safety, accurate weather forecasts, and disaster warning.

#### Self-powered hydrological and gas sensors

**Figure 5:**
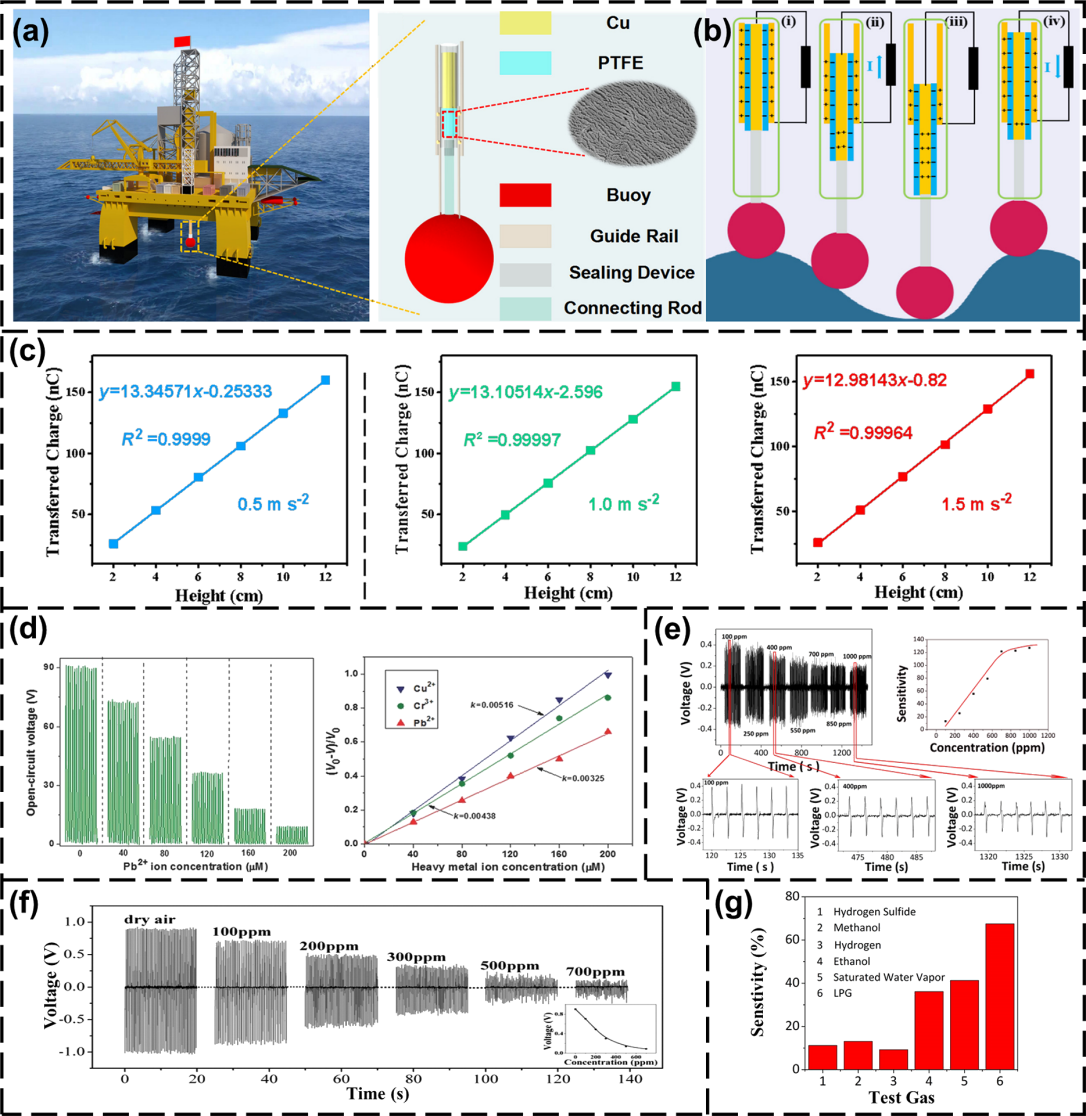
Self-powered hydrological and gas sensor for environmental data collection. (a) The appearance structure of TOSS [12]. Figure 5a–c was reproduced with permission from [12] (for permission, please see the caption of Figure 1d). (b) The principles of TOSS to obtain sea wave information [12]. (c) Relationship between wave height and transferred charges under different accelerations [12]. (d) Output voltage of the self-powered tribonanosensor under different heavy metal ion concentrations [16]. Figure 5d was reproduced from [16]. Copyright © 2016 WILEY‐VCH Verlag GmbH & Co. KGaA, Weinheim. Used with permission from Zhaoling Li et al., “Triboelectrifcation‐Enabled Self‐Powered Detection and Removal of Heavy Metal Ions in Wastewater”, Advanced Materials, John Wiley and Sons. (e) Output voltage of the self-powered sensor under different H_2_S gas concentrations at room temperature. The sensitivity *S* varies with H_2_S gas concentration [18]. Figure 5e was reproduced from [18] (for permission, please see the caption of Figure 1f). (f) Piezoelectric output voltage relationship of the device when In_2_O_3_/ZnO NW is exposed to dry air and H_2_S gas of various concentrations at room temperature [20]. Figure 5f was reproduced with permission from [20], Copyright 2014 American Chemical Society. (g) Sensitivity of ZnSnO_3_/ZnO NW-based SPGS to H_2_S, H_2_, ethanol, methanol, LPG, and saturated moisture at 4000 ppm [21]. Figure 5g was reproduced with permission from [21], Copyright 2015 American Chemical Society.

Zhang et al. reported a triboelectric ocean-wave spectrum sensor (TOSS) [12]. The structure of the device is shown in Figure 5a. The TOSS collects wave data from a buoy. While ocean waves move the buoy up and down, charges are generated in a TENG, as shown in Figure 5b. The generated charges are linearly correlated with the wave heights, as shown in Figure 5c. From this linear relationship, the wave height data can be obtained, including period and speed of waves.

Environmentally friendly, portable, easy-to-deploy, and low-cost TENGs are novel devices for water quality monitoring. Zhou et al. proposed the use of waste materials to make an arc-shaped TENG (AS-TENG) [15]. The AS-TENG can provide energy for a pH sensor in water. The system can trigger an alarm when the pH value is lower than 5. Lee et al. proposed a based Hg^2+^ ion sensor based on ZnO nanowires and carbon nanotubes for detecting toxic pollutants [17]. The ZnO nanowire (NW) array acted as power source. When Hg^2+^ ions were detected, the system powered a light-emitting diode (LED). Li et al. designed a self-powered heavy metal ion triboelectric nanosensor [16]. By adding three ligand molecules to the surface of nanoporous anodic aluminum oxide, the detection sensitivity for Cu^2+^, Pb^2+^, and Cr^3+^ reached 0.005 × 10^−6^, 0.003 × 10^−6^, and 0.004 × 10^−6^ M, respectively. The self-powered tribo-nanosensor generates different output voltages according to the ion concentration, as shown in Figure 5d. The TENGs are a low-cost and environmentally friendly solution for detecting heavy metal ions. The kinetic energy generated by the flow of waste water is converted into electricity through a water-driven triboelectric nanogenerator (WD-TENG). Thus, heavy metal ions in waste water can be removed without external power consumption. The self-powered sensor collects water quality information such as ion concentration in the water as a data source for hydrological analysis.

PENG/TENG-based pressure sensors can accurately sense pressure changes through the output response signal. Gas molecules can be adsorbed on the surface of piezoelectric/triboelectric materials, causing changes in the carrier density [95]. Thus, the gas concentration can be obtained from the output voltage of the PENG/TENGs. The output of a PENG based on ZnO NWs is largely influenced by the surface carrier density on the surface of the nanowires. The adsorption of gas molecules can change the surface carrier density by the shielding effect, so the output of the sensor is very sensitive to the gas concentration. Compared with traditional metal oxide semiconductor (MOS) gas sensors, the PENG/TENG-based self-powered gas sensor has a lower power consumption, requires no heating, and exhibits high stability and high sensitivity.

In 2013, Xue et al. proposed a ZnO NWs PENG-based self-powered gas sensor [18]. The response of the unpackaged PENG sensor was studied under exposure to oxygen (O_2_), hydrogen sulfide (H_2_S) gas, and water vapor. The sensitivity to H_2_S gas was as low as 100 ppm. The design of the whole self-powered sensor system consists of three parts. A ZnO NW array as the piezoelectric energy generation module, Ti foil and Al layer as electrodes, and kapton boards as supporting frame. Under the same deformation conditions, the sensitivity *S* of a PENG/TENG can be simply defined as:

[1] $$S\left[\%\right]=\frac{V_{\text{a}}-V_{\text{g}}}{V_{\text{g}}}\times 100\%,$$

where *V*_a_ is the piezoelectric output voltage in dry air (the concentration of the gas to be measured is 0 ppm), and *V*_g_ is the piezoelectric output voltage during exposure to the test gas. The sensitivity of above ZnO-based self-powered sensor is 127.3% under 1000 ppm H_2_S. The piezoelectric output of sensor decreased with increase of the concentration of the tested gas, as shown in Figure 5e. In 2016, a self-powered gas sensor based on a NiO/ZnO heterojunction nanowire array showed high sensitivity and fast response [19]. At room temperature, the piezoelectric voltage of the NiO/ZnO NW array was reduced from 0.388 V (in dry air) to 0.061 V (at 1000 ppm H_2_S), and the response was approximately 10 times that of naked the ZnO NW array. An In_2_O_3_/ZnO heterostructure was prepared the sensitivity of which was 925% [20]. Figure 5f shows the piezoelectric output voltage of In_2_O_3_/ZnO NW array exposed to dry air and H_2_S gas of various concentrations at room temperature. Compared with the gas adsorption reaction of ZnO material, the conversion of In_2_O_3_/ZnO to In_2_S_3_/ZnO has a stronger regulating effect on the piezoelectric filtration of free carriers.

A ZnO-based self-powered gas sensor (SPGS) can be used to detect H_2_S, NH_3_ [96–97], ethanol [91,98–100], CO_2_ [101–102] and other gases [103–105]. A ZnSnO_3_/ZnO NW-based PENG was used to detect liquefied petroleum gas (LPG) with high sensitivity, selectivity, and reliability [21]. The sensitivity of ZnSnO_3_/ZnO (1 h) NWs in 4000 ppm H_2_S, H_2_, ethanol, methanol, LPG, and saturated water vapor was measured. The sensitivity of ZnSnO_3_/ZnO NW for LPG is much higher than for other gases, as shown in Figure 5g. Modaresinezhad et al. used a DC nanogenerator based on ZnO nanosheets as room-temperature self-powered humidity sensor [103].

This new type of self-powered gas sensors will be an important development direction for the next generation of gas sensors [95]. In addition, the above self-powered sensors show that they are easier to deploy and consume less power than traditional MOS gas sensors. Because of this, self-powered sensors can be deployed in large numbers, and a large number of self-powered sensors will generate a massive amount of data. Through big data and AI analysis the back-end data processing capability can be enhanced. Moreover, AI technology can also enhance the performance of sensor.

#### Traffic sensors

**Figure 6:**
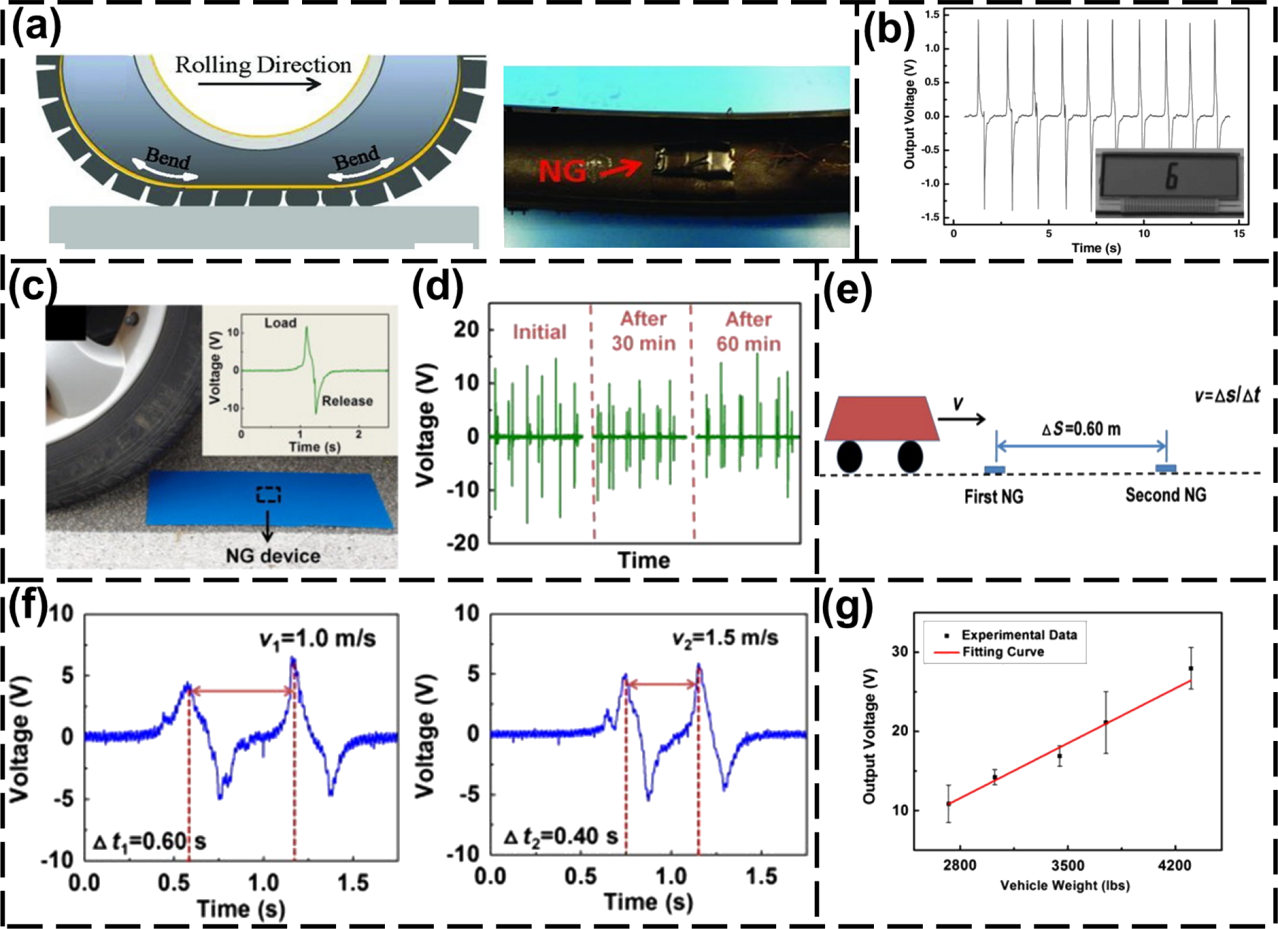
NG-based self-powered traffic sensor. (a) PENG attached to a tire with adhesive tape [5]. Figure 6a,b was reproduced from [5]. Copyright © 2011 WILEY‐VCH Verlag GmbH & Co. KGaA, Weinheim. Used with permission from Youfan Hu et al., “A Nanogenerator for Energy Harvesting from a Rotating Tire and its Application as a Self‐Powered Pressure/Speed Sensor”, Advanced Materials, John Wiley and Sons. (b) The output voltage of a PENG and the LCD powered by the PENG when the tire rotates [5]. (c) The voltage output after the sensor is pressed by the wheel once [3]. Figure 6c–g was reproduced from [3], (for permission, please see the caption of Figure 1e). (d) After being rolled over by wheels for an extended period of time, the output voltage of the TFNG changes only slightly [3]. (e) Schematic diagram of measuring speed via a TFNG [3]. (f) Voltage output of the TFNG as a function of the time at different vehicle speeds [3]. (g) Relationship between vehicle weight and output voltage of the TFNG [3].

PENGs are also applicable in intelligent transportation systems. Self-powered vehicle sensors based on PENGs/TENGs can collect the signal of force changes when the vehicle status changes. In 2011, Hu et al. [5] proposed a flexible PENG that was attached to a vehicle tire. During rotation of the tire, the electrical pulses were generated by the PENG. These electrical signals can be used as sensor outputs to calculate the vehicle speed and provide energy for an external system. The PENG consisted of top and bottom Cr/Au electrodes, ZnO NWs on the electrodes and a flexible polyester substrate. Figure 6a shows the PENG fixed to the inner surface of the tire with adhesive tape. The output voltage of the PENG changed with the deformation of the tire during rolling. Figure 6b shows the output open-circuit voltage during tire movement. Speed information of the vehicle can be obtained from the output voltage.

Speed and acceleration sensors based on PENGs/TENGs were used to detect the driving status. Acceleration information can be obtained from the tire movement of the vehicle. Zhang et al. proposed a self-powered acceleration sensor based on liquid-metal triboelectric nanogenerator (LM-TENG), which can directly detect horizontal and vertical accelerations [6]. When a vehicle crashes, the force and position data of the vehicle can be obtained through the acceleration sensor. This acceleration sensor can be used to detect the acceleration of a vehicle climbing uphill. This application can provide data support for the vertical motion information for future 3D car navigation. A driving safety warning system can detect the tire pressure to determine whether the vehicle has too high or unbalanced load [7]. To detect the state of wheels at high friction and at high speed, sensors based on a harsh-environmental TENG (he-TENG) can be included in a self-powered smart brake system. TENG-based vehicle sensors can collect data on driving habits, such as the frequency of using brake pedal and accelerator pedal under different road conditions [8]. Thus, TENG-based warning and brake systems may be designed for future autonomous vehicles.

Intelligent transportation is emerging with the development of IoT technology [106]. Traditional sensor networks are powered by batteries or energy grids. Thus, the deployment and maintenance of sensors will bring challenges for intelligent transportation systems. PENGs/TENGs can gain energy from vibrations without the need for an energy grid. In 2013, Lin et al. [3] proposed a transparent and flexible PENG (TFNG) based on a flexible polydimethylsiloxane (PDMS) substrate and ZnO NWs. It could be deployed on the road for speed and weight detection, as well as collecting mechanical energy from rolling wheels to power a LCD. Figure 6c shows the output voltage signal when the wheel runs over the TFNG. The output of TFNG has good durability, as shown in Figure 6d. The principle of measuring vehicle speed and weight is shown in Figure 6e. Two TFNGs with a distance of Δ*S* = 0.6 m were placed on the road. When the vehicle passed the TFNGs, two peak waveforms were generated. The speed can be calculated from the peak time difference Δ*t* between two waveforms: *v* = Δ*S*/Δ*t*. Figure 6f shows the output voltage at vehicle speeds of 1 and 1.5 m/s. The output voltage of the TFNG is proportional to the weight of the vehicle, as shown in Figure 6g. A TENG-based self-powered sensor can also be deployed to detect bridge vibrations [68]. When the bridge vibrations are within the safety limit, the TENG generates AC signals for power supply and analysis of vibration characteristics. When the vibration exceeds a threshold, the output signal becomes a DC signal and an alarm is triggered.

To sum up, self-powered sensors based on NGs have great prospects as intelligent traffic sensors, and they show excellent performance in traffic monitoring. Abundant sensor data can establish smart transportation and improve driving safety and convenience. Also they are the basis of data for autonomous driving.

#### Key challenges and potential solutions for future self-powered sensors

In the future, NG-based self-powered sensors can be used in the collection of external environment data and human physiological data. NG-based self-powered sensors are good candidates for “smart dust”, which requires independent continuous work and the collection massive data [107]. We propose several key challenges and directions for the future development of NG-based self-powered sensors:

**1. Application-specific integrated circuits (ASICs) designed for TENG sensors.** The energy, such as mechanical energy, chemical energy, thermal energy, and light energy, that can be gained from the environment maybe limited and random. TENG sensors require ASICs with fast power-up, high performance and low energy consumption. The key properties of the ASICs should be ultra-short power-on time and ultrafast processing, while the performance should be higher than that of traditional ICs. Thus, the ASICs will have a smaller average power consumption. It is necessary to design energy management, information processing, and communications ASICs for TENG applications.

**2. The embedded operating system based on adaptive energy unit.** The random energy from the environment makes it difficult for TENG sensors to work continuously. A low-power high-performance sampling algorithm could collect the main data. It is necessary to develop new theories and algorithms for TENG sensors. The embedded operating system would be based on these new theories and algorithms, which effectively control information and energy flows.

**3. AI for TENG-based self-powered sensors.** The bottleneck of the self-powered systems is the quality of data due to the randomness of the energy from the environment. AI algorithms could learn the complete information features and complete the missing information in the TENG sensor data. AI algorithms for energy management are also an important topic for TENG applications.

**4. New communication systems designed for TENG sensors.** TENG-based self-powered sensors have great advantages for large-scale deployment. The transmission of massive data will rise challenges. Efficient and low-power communication systems are necessary. Another solution is compressing data for communication, which reduces the scale of data transmission.
